# Novel phthalocyanines activated by dim light for mosquito larva- and cell-inactivation with inference for their potential as broad-spectrum photodynamic insecticides

**DOI:** 10.1371/journal.pone.0217355

**Published:** 2019-05-29

**Authors:** Shin-Hong Shiao, Shih-Che Weng, Liqiang Luan, Maria da Graça H. Vicente, Xiong-Jie Jiang, Dennis K. P. Ng, Bala Krishna Kolli, Kwang Poo Chang

**Affiliations:** 1 Department of Parasitology, National Taiwan University, Taipei, Taiwan; 2 Department of Chemistry, Louisiana State University, Baton Rouge, LA, United States of America; 3 Department of Chemistry, the Chinese University of Hong Kong, Shatin, N. T., Hong Kong; 4 Department of Microbiology/Immunology, Chicago Medical School/Rosalind Franklin University of Medicine & Science, North Chicago, IL, United States of America; Massachusetts General Hospital, UNITED STATES

## Abstract

Mosquitoes are significant vectors, responsible for transmitting serious infectious diseases, including the recent epidemics of global significance caused by, for example, Zika, Dengue and Chikungunya viruses. The chemical insecticides in use for mosquito control are toxic and ineffective due to the development of resistance to them. The new approach to reduce mosquito population by releasing genetically modified males to cause female infertility is still under environmental safety evaluation. Photodynamic insecticides (PDI) have long been known as a safe and effective alternative by using dyes as the photosensitizers (PS) for activation with light to generate insecticidal singlet oxygen and reactive oxygen species. This approach warrants re-examination with advances in the chemical synthesis of novel PS, e.g. phthalocyanines (PC). Nine PC were compared with five porphyrin derivatives and two classic PS of halogenated fluoresceins, i.e. cyanosine and rose bengal experimentally for photodynamic treatment (PDT) of the larvae of laboratory-reared *Aedes* mosquitoes and their cell lines. Groups of 2^nd^ instar larvae were first exposed overnight to graded concentrations of each PS in the dark followed by their exposure to dim light for up to 7 hours. Larvae of both experimental and control groups were examined hourly for viability based on their motility. Monolayers of mosquito cells were similarly PS-sensitized and exposed briefly to light at the PS-specific excitation wavelengths. Cell viability was assessed by MTT reduction assays. Of the 16 PS examined for photodynamic inactivation of the mosquito larvae, effective are three novel PC, i.e. amino-Si-PC1 and -PC2, anilinium Zn-PC3.4, pyridyloxy Si-PC14 and two porphyrin derivatives, i.e. TPPS2 and TMAP. Their EC_50_ values were determined, all falling in the nanomolar range lower than those of rose bengal and cyanosine. All PS effective *in vivo* were also found to dose-dependently inactivate mosquito cells photodynamically *in vitro*, providing cellular basis for their larvicidal activities. The present findings of novel PC with effective photodynamic larvicidal activities provide fresh impetus to the development of PDI with their established advantages in safety and efficacy. Toward that end, the insect cell lines are of value for rapid screening of new PC. The optimal excitability of PC with insect-invisible red light is inferred to have the potential to broaden the range of targetable insect pests.

## Introduction

Photodynamic therapy or treatment (PDT) is referred to the use of dyes as photosensitizers (PS) for light excitation to produce biocidal oxidative radicals in the presence of oxygen. PDT has long been used clinically for treating patients with solid tumors, certain skin diseases, infection and other medical conditions [1]. The application of PDT to control insect pests has been studied since the early 1900’s [2]. From 1987 to1995, the American Chemical Society published three symposium volumes devoted to light-activated pesticides [3–5]. Since then, follow-up investigations have been sporadic, as summarized in a handful of reviews [6–8]. Halogenated fluoresceins and some natural dyes were the classic PS used in the early work for experimental and field trials of PDT against various insects, including mosquito larvae and Mediterranean fruit flies. Industrial application (PhotoDye International, Inc) progressed to the stage of using aerial spray of dye mixtures (xanthenes) (Red Dye #28 and Yellow Dye #8) or SureDye [9] in attempt to control the latter pest of agricultural importance.

While photodynamic insecticides (PDI) have not gained extensive attention thereafter, there is clear evidence, indicative of their safety and efficacy. The safety of PS is self-evident, considering their household use as food, drug, cosmetic and fabric dyes. Large magnitude of environmental and human safety of PS, like porphyrins and phthalocyanines (PC) have long been experimentally proven and thoroughly addressed [10–11]. The most significant, but not well-recognized is the efficacy of PDT in their aversion to select organisms for resistance. This is based on the well-known mode of PDT action: neither PS nor light alone is biocidal, thereby exerting no pressure to select for resistance, while their combination results in the production of powerful cytotoxic oxidative radicals, which attack too many vital molecules simultaneously for the targets to develop resistance. PDI thus differs from the single-target insecticides, to which resistance arises inevitably and rapidly as a recurrent problem [12].

Application of PDI to control mosquitoes is thus highly desirable, e. g. *Anopheles* and *Culex*, which transmit malaria, and filariasis and West Nile fever, respectively. Moreover, *Aedes* spp. transmit arboviruses, which cause Dengue, Zika, Chikungunya and Yellow Fever, responsible for epidemic outbreaks of severe diseases in the tropical/subtropical world today. Aside from the development of conventional chemical pesticides and its integration with biological controls for pest managements, the new strategy under study to control these vectors is the genetic approach that is to develop genetically modified (GM) male mosquitoes for release to cause female infertility, thereby reducing the vector population in the field [13–14]. Environmental safety evaluations of this approach are still pending for its implementation. The larval stages of all mosquitoes are aquatic and thus receptive to water-soluble PS for PDT. Mosquito larvae of the disease transmitting *Aedes*, *Culex* and *Anopheles* were indeed among the first insect target for investigation using sunlight- activated fluorescein-based PS, i.e. erythrosine and rose bengal [2]. A variety of different PS have been examined since then for activation by solar or artificial light against these and other mosquito larvae, e.g. marigold alpha-terthienyl [15–17], rose bengal in comparison to porphyrins [18], Phytoalexins phenalenones [19] and cationic water-soluble meso-substituted porphyrins [20–21].

Here, we report the results of our studies, which were started initially by comparing two halogenated fluoresceins (rose bengal/cyanosine) with protoporphyrin IX and two phthalocyanines (AlPhCl and novel PC3) for their PDT activities against mosquito larvae of different species *in vivo* and cultured cells of *Aedes albopictus* clone C6/36 (ATCC CRL-1660) *in vitro* [22–23]. The preliminary results obtained are encouraging, leading us to examine seven additional novel PC and five porphyrin derivatives in comparison to the two halogenated fluoresceins. These PS were compared for their relative PDT activities against *Aedes* larvae *in vivo* and mosquito cells from a different source *in vitro*. Five of the 16 PS examined, i.e. three novel PC and two porphyrin derivatives, were found to mediate photodynamic larvicidal activities favorably in comparison to the halogenated fluoresceins. The five PS were taken up by insect cells and PDT-inactivated them *in vitro*, accounting for their larvicidal activities and suggestive of the potential use of this *in vitro* system for screening PDI. The addition of PC as new arsenals to PDI is envisioned from their excitability by insect-invisible red light to have the potential to extend the range of their targetable insects.

## Materials and methods

### Chemicals

**Fig 1:**
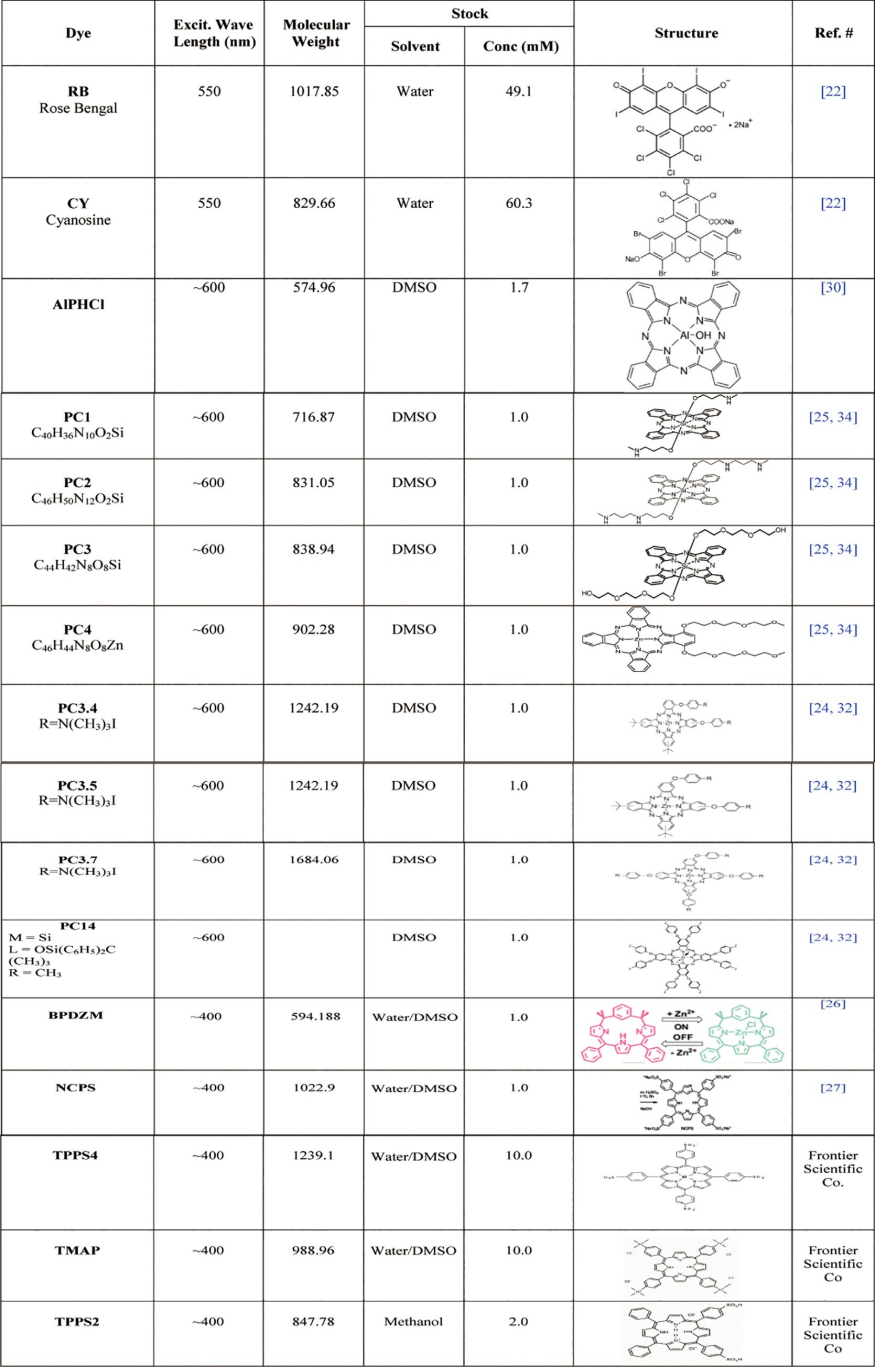
Physical and chemical properties of photosensitizers used in the present study. lPhCl: Aluminum phthalocyanine chloride (Sigma). PC1-2: Amino-phthalocyanines; PC3-4: triethylene glycol-substituted Zn(II)-phthalocyanines. PC3.4–3.7: Anilinium Zn-phthalocyanines. PC14: Pyridyloxy Si-phthalocyanine. BPDZM: M-Benziporphodimethene. NCPS: Meso-tetrakis(p-sulfonatophenyl)N-confused porphyrin tetrasodium. TPPS4: Meso-tetra(4-sulfonato-phenyl)porphineTetrasodium. TMAP: Meso-tetra(4-n,n,n-trimethylanilinium)porphine tetrachloride. TPPS2: Meso-Tetraphenylporphinedisulphonic acid dihydrochorid.

The nine phthalocyanines, five porphyrin derivatives and two halogenated fluoresceins examined in this study for comparison are listed in Fig 1. All novel PCs were synthesized and HPLC-purified [24–25]. Porphyrin derivatives [26–27] were kindly provided by colleagues from commercial sources (Frontier Scientific Co.).

### Mosquitoes

*Aedes aegypti* UGAL/Rockefeller strain was reared as described [28, 29]. Briefly, adults were fed with 10% sucrose solution and maintained in an institutionally approved insectarium under the ambient conditions of 28°C, relative humidity of 75–80% and a light/dark cycle of 12:12 h. Feeding of fertilized female mosquitoes on mice was carried out as follows: Male ICR (Institute of Cancer Research, USA) mice, each ~35 gram in bodyweight or ~8 weeks old, were obtained from the Laboratory of Animal Center at National Taiwan University (Taipei, Taiwan) and handled by trained personnel for this study with the approval of the National Taiwan University College of Medicine and College of Public Health Institutional Animal Care and Use Committee (ID #20100268). Three ICR mice were maintained in one individually ventilated cage of 50 cm x 20 cm x 30 cm in size with regular mouse food (MFG, Oriental Yeast Co. Ltd). The temperature was set at 25°C with humidity of 30–50% and a light/dark cycle of 12:12 h. The water bottles were changed daily. The ICR mice were each anesthetized by intraperitoneal injection at a dosage of 250 mg/Kg with Avertin, consisting of 2.5 gram of 2,2,2 Tribromoethanol and 5 ml 2-methyl-2-butanol (amylene hydrate, tertiary amyl alcohol) in 200 ml distilled water. Female mosquitoes were collected 3–5 days post eclosion and placed in group of 100 in a screened mosquito cage. An Avertin-anesthetized ICR mouse was placed on top of each cage, allowing the mosquitoes therein to take blood meals. All female mosquitoes were engorged in ~1 hour, except very few, which were not expected to lay eggs and thus removed.

### Mosquito cell lines

*Aedes* mosquito cells of the ACT10 (*A*. *aegypti*) or ACT15 (*A*. *albopictus*) lines (courtesy of Dr. Cindy L Goodman, USDA-ARS, Colombia, MO) were cultured at 25°C in Schneider’s Medium+10% HIFBS as monolayers in 25 cm^2^ TC flasks and cryopreserved in liquid nitrogen. Cryopreserved cells were thawed and grown as described before use.

### Photodynamic therapy or treatment (PDT)

**Fig 2:**
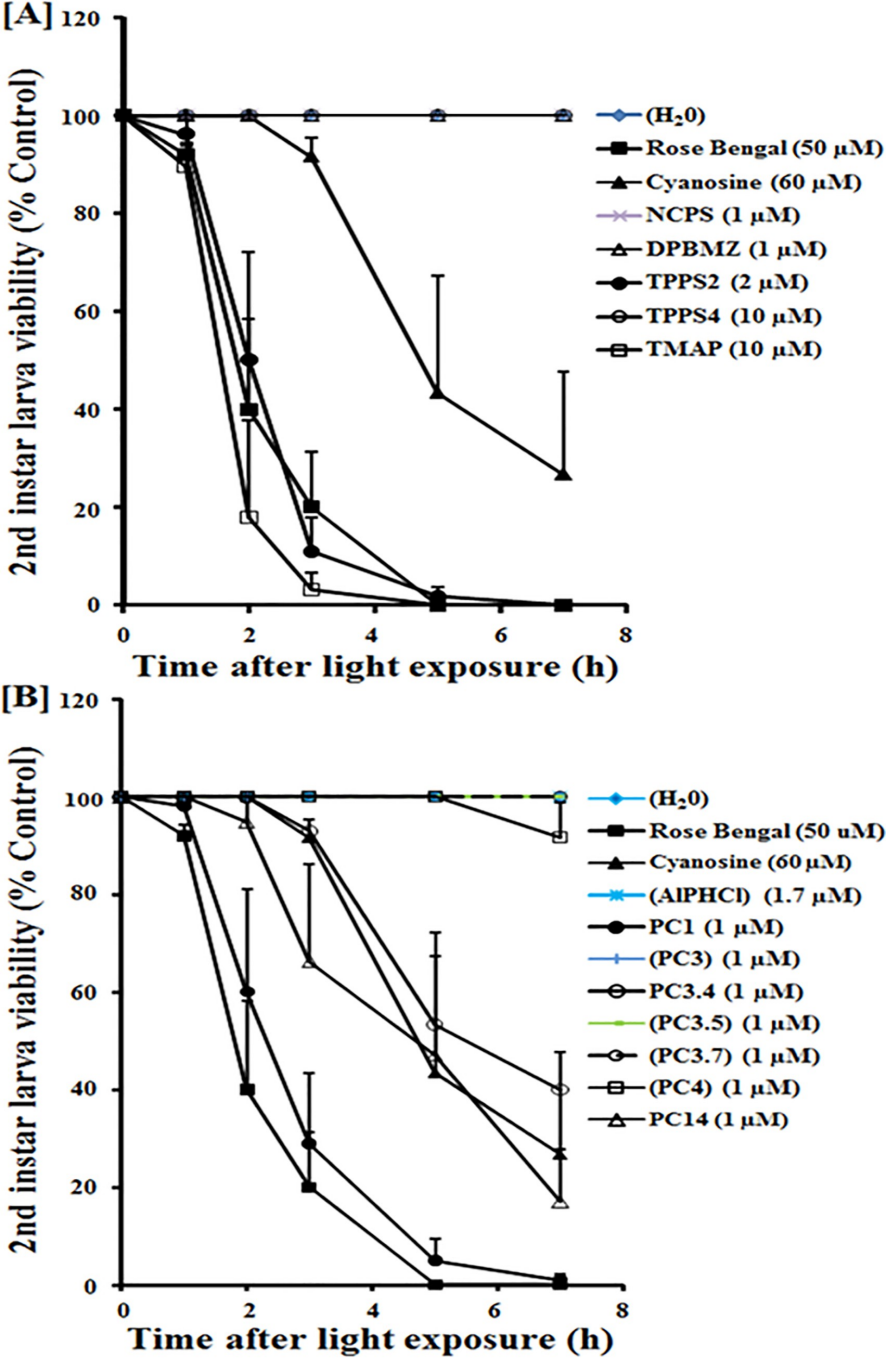
**Sensitivity of 2**^**nd**^ **instar *Aedes aegypti* mosquito larvae to light-induced inactivation mediated by porphyrins [A] and phthalocyanines [B] at high concentrations.** See Materials and Methods for experimental details. Briefly, groups of ~20 2^nd^ instar larvae were exposed in the dark overnight to the photosensitizers (PS) at the concentrations as indicated. For each PS, one set of PS-exposed larvae was left in the dark and the other set exposed to white-light. Dead and live larvae were tallied hourly for 7 hours in all sets. Viability of the larvae was determined by visual inspection for their loss of motility and presented in % as the ratio of dead larvae in light-exposed versus dark conditions. Data presented represent results from three or more independent experiments. Shown here are time-dependent larval immobilizations by light after exposure to individual photosensitizers at 10^−3^ dilutions of the stock solutions. Individual photosensitizers (PS) were designated by different symbols in each graph. Refer to **Fig** 1 for PS details. The final concentration is given in brackets after each PS. **Note**: Not shown are the data for PC2, which produced essentially the same results as those of PC1. *** *p* values < 0.001 in comparison to the control larvae in water at the end point of 7 hour.

**Fig 3:**
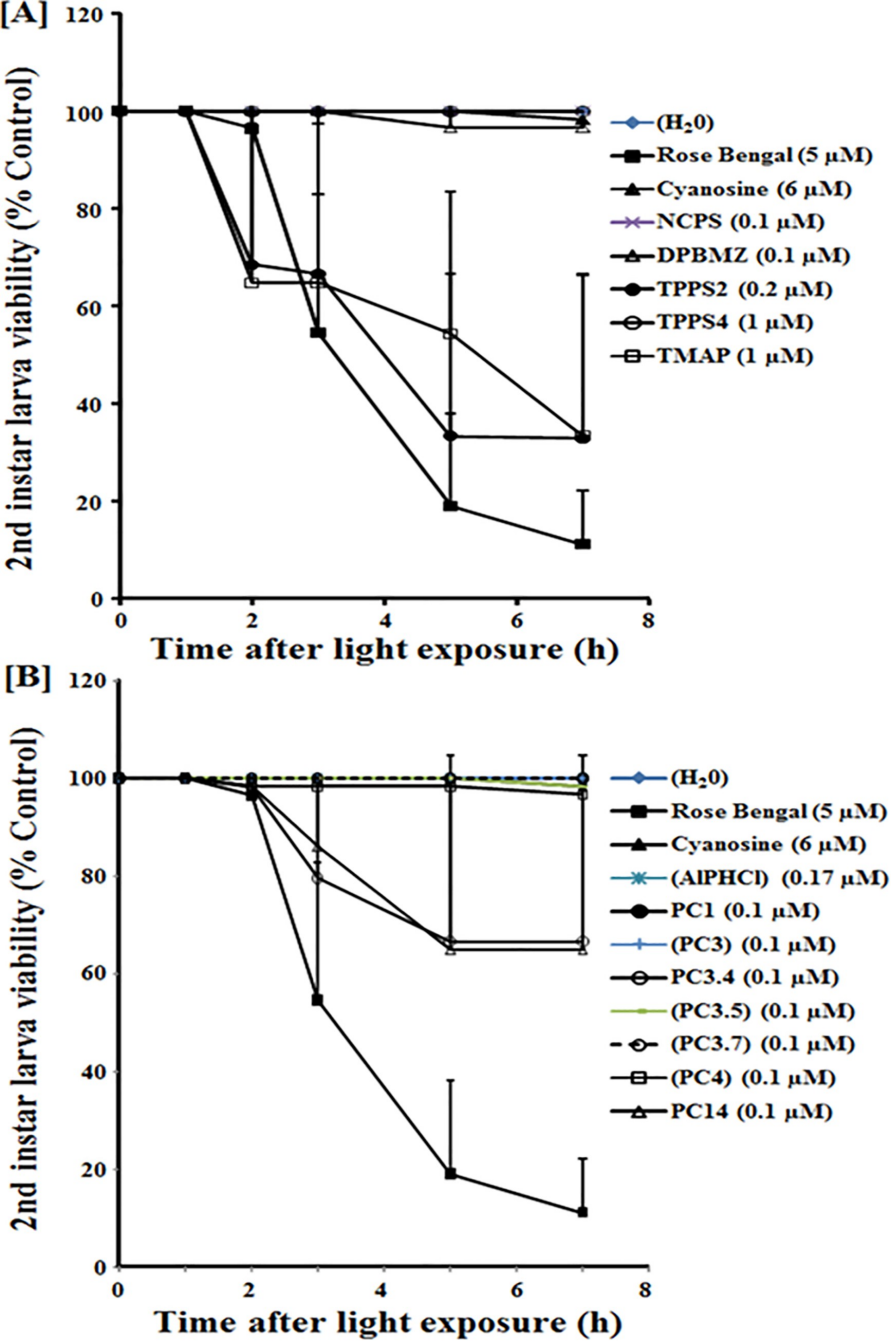
**Sensitivity of 2**^**nd**^ **instar *Aedes aegypti* mosquito larvae to light-induced inactivation mediated by porphyrins [A] and phthalocyanines [B] at low concentraitons.** See legend to Fig 2 for experimental and other details, which are identical for this set of experiments, except for using 10^−4^ dilutions of the stock solutions.

*In vivo* PDT assay for larvicidal activities of the listed PS was based on the general procedures with modifications from those previously developed for other eukaryotes, i.e. mammalian cells and protozoa [30–35]. Briefly, each PS was tested initially in two concentrations at 1/1,000 (Fig 2) and 1/10,000 (Fig 3) dilutions of its stock solution (see Fig 1 for the concentrations of individual PS stock solutions and in-graph legends of Figs 2 and 3 for the final concentrations used). The stock solutions varied in concentrations with different PS due to the differences in their solubility in the solvents, i.e. water, methanol or DMSO. The organic solvents (methanol, DMSO) at the highest PS concentrations used was ≤ 0.1%, which was pre-tested alone and found to have no larvicidal activity. Larvae in group of 20 per dish were exposed to each PS in 5 ml of tap water. All samples were wrapped with light-impervious foil and incubated overnight for PS uptake in the dark. After 16 hours of PS-loading, one of the duplicate dishes was un-wrapped and uncovered for exposure to white fluorescent light from the top (1–2 J/cm^2^). A constant ambient temperature was maintained at 27°C for the duration of the illumination. The remaining dish of each set remained foiled-wrapped to serve as the dark control under otherwise the same ambient conditions. Larvae of all experimental and control groups were visually checked hourly for up to 7 hours and the number of immobilized larvae recorded. The loss of mobility of mosquito larva has been established as a simple method for reliable determination of their viability [36]. All larvae were found to remain motile and thus viable under the dark conditions.

*In vitro* PDT assay of mosquito cells was carried out to assess their uptake of PS for light-mediated inactivation/disintegration. *Aedes* mosquito cells of both species were grown as monolayers under the described conditions for exposure to selected PS at graded concentrations of 0, 0.01, 0.1 and 1 μM in the dark for ~16 hours. Adherent cells were then loosened by repeated gentle pipetting and reseeded in triplicate, each at 5 X 10^5^ cells/well in 24-well culture plates. The plates with PS-exposed cells were divided into two groups: one group kept in the dark and the other light-exposed for 20–30 min (1–2 J/cm^2^). The PC-loaded cells were exposed to red light (λ_max_ = ~600 nm) from the bottom of the plates, and porphyrin-exposed cells to longwave UV (λ_max_ = 366 nm) from the top of the plates with lid off in the biosafety cabinet. After further incubation overnight, cell samples from all groups were examined for their integrity and uptake of PS by phase contrast and fluorescent microscopy, as described previously [30–35]. The remaining cell samples from all groups were assessed in triplicate for their viability by MTT reduction assays [33, 35].

### Determination of EC_50_ values

The EC_50_ values of representative PS for larvicidal PDT were initially estimated tentatively from the PS concentration-versus-larva survival plots with data taken at the end point of the light exposure for 7 hours (Figs 2 and 3). The EC_50_ values were subsequently determined more rigorously in three independent experiments for the five effective PS, each in serial dilutions of three concentrations of 0.01, 0.1 and 1 uM. The EC_50_ values of selected PS for PDT of mosquito cells were similarly derived from PS concentration-versus-MTT cell viability plots.

### Data analysis/presentation

All *in vivo* studies were repeated as independent experiments for >3 times. All *in vitro* experiments were repeated at least twice and, in most cases, three times. The results of *in vitro* studies obtained were comparable among repeat experiments. The data presented represent the means with standard errors of the values obtained in triplicate for individual samples from representative experiments. Data analyses were done for the 7 hour end point of larva PDT in Sigmaplot 12 using one-way RM ANOVA with pairwise multiple comparison of Holm-Sidak method. MTT data analyses for cell viability were performed by pairwise data comparison with two-tailed Student t tests in GraphPad Prism version 5. *P* values of <0.05 were considered as significant.

## Results

### Instar-dependent PDT larvicidal activities

This was evident by comparing the sensitivity of 2^nd^, 3^rd^ and 4^th^ instar larvae of the mosquitoes for their immobilization by effective PS-mediated PDT. The 2^nd^ instar larvae were found most sensitive, as shown in representative data with the five effective PS (S1A–S1E Fig). Two PS, e.g. rose bengal and PC1 were effective against older larvae, but manifested only after exposure to light for 7 hours at the end point (S1A and S1B Fig). Data were thus obtained from further studies with 2^nd^ instar larvae, as presented in detail below.

### PS- and time-dependent larvicidal activities

Figs 2 and 3 shows the results for time-dependent 2^nd^ instar larvicidal activities obtained with listed porphyrin derivatives ([A]) and phthalocyanines (PC) ([B]), each at 1/1,000 (Fig 2) and 1/10,000 (Fig 3) dilutions of their respective stock solutions (see Fig 1. Note: The concentrations of the stock solutions vary with different porphyrin derivatives and halogenated fluoresceins, while those of PC are identical at 1 mM). Negative controls without PS (H_2_O) and those with rose bengal and cyanosine were included for reference. Larvicidal activities based on larva loss of motility with time of light exposure are presented as % of dark control.

Of the five porphyrin derivatives examined, TMAP (open square) and TPPS2 (solid circle) were larvicidal at both dilutions of the stocks used, as indicated by the progressive increase in the number of immotile or dead larvae with time of illumination (Figs 2A and 3A). Both PS were more effective than rose bengal (Solid square) and cyanosine (solid triangle), taking into account the differences in their final concentrations used (Figs 2A and 3A, figure legends). Cyanosine was least larvicidal in comparison to the effective PS. The remaining three porphyrin derivatives (NCPS, DPBMZ, TPPS4) showed no larvicidal activity at both dilutions of their stocks (Figs 2A and 3A).

Of the nine PC examined, PC1 (solid circle), PC2 (Not shown, data similar to PC1), PC3.4 (Open circle) and PC14 (Open triangle) were larvicidal, more evident at 1:1,000 dilution (Fig 2B) than at 1:10,000 dilution (Fig 3B) with reference to rose bengal (solid square) and cyanosine (solid triangle). Under the same conditions, the remaining five PCs were ineffective (AlPhCl, PC3, PC3.5, PC3.7, PC4). Large variabilities among independent experiments are noted, especially at the lower dilutions. This is not unexpected due to a number of uncontrollable factors inherently associated with batch-to-batch differences.

### Uptake of effective PS by *Aedes* cell lines and their photo-inactivation *in vitro*

**Fig 4:**
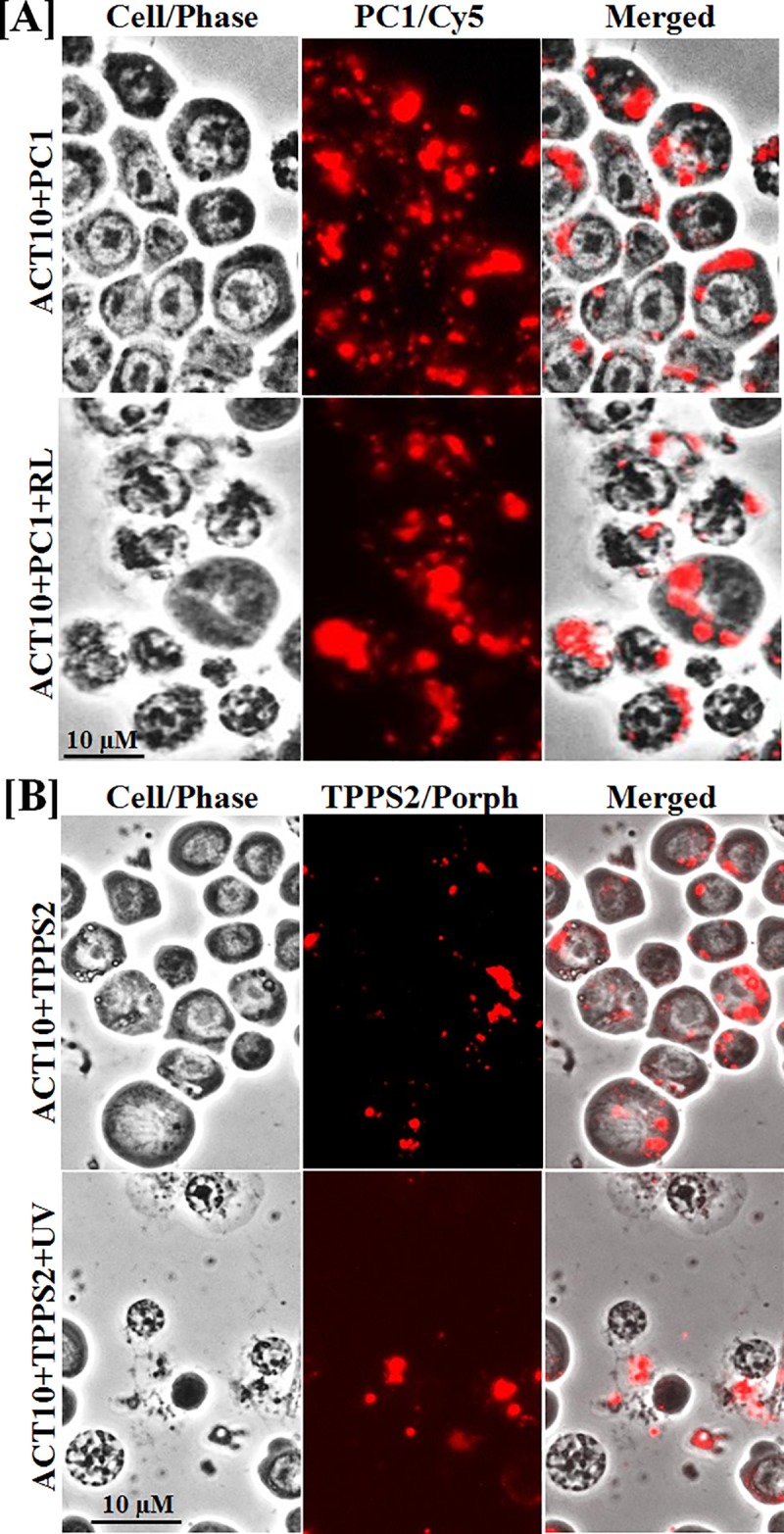
**Uptake of representative phthalcyanine PC1 [A] and porphyrin TPPS2 [B] by *Aedes* cells effective to mediate their photo-inactivation and disintegration *in vitro*.** See Materials and Methods for experimental details. Briefly, ACT10 cells were exposed *in vitro* to the photosensitizer (PS) in the dark overnight. One set was kept in the dark (Upper row: [A] ACT10+PC1 and [B] ACT10+TPPS2), while the other set was exposed to light at the PS-specific excitation wavelengths (lower row: [A] ACT10+PC1+RL and [B] ACT10+TTPS2+UV). Cell/Phase, Phase contrast microscopy to show cell integrity; PC1/Cy5, Cy5 filter set used to show PC1 fluorescence; TTPS2/Porph, Porphyrin filter set used to show TTPS2 fluorescence. Merged, phase contrast and fluorescence images merged to show uptake of both PS by the cells. **Note:** disorganization/disintegration of PS- and light-exposed cells in both cases (lower rows of [A] and [B]).

By phase contrast and fluorescent microscopy, ACT10 and ACT15 *Aedes* cells were found to take up the PS, which mediated *in vivo* light-activated larvicidal activities, but not ineffective PS in most cases. This is illustrated by the images from two effective representative PS, i.e. phthalocyanine PC1 and porphyrin derivative TPPS2 (Fig 4). Incubation of the cells with both PS in the dark (Fig 4 upper row: [A] ACT10+PC1; [B] ACT10+TPPS2) showed that they were morphologically intact (Cell/Phase), but contained fluorescent PC1 (PC1/Cy5) and TPPS2 (TPPS2/Porph) in their cytoplasm (Merged). Exposure of these PS-loaded cells to light (Fig 4. Lower row: [A] ACT10+PC1+RL and [B] PCT10+TPPS2+UV) resulted in their disintegration (Cell/Phase), leaving PS in degenerated cells or scattered among cell debris (Merged). Under similar experimental conditions, the other three larvicidal PS produced similar results, i.e. PC3.4, PC14 and TMAPS, whereas all ineffective PS (three porphyrin derivatives and five phthalocyanines) were not taken up at all or marginally taken up by the insects cells and produced little or no cytolysis after light exposure (not shown).

### PS concentration-dependent photo-inactivation of *Aedes* cells *in vitro*

**Fig 5:**
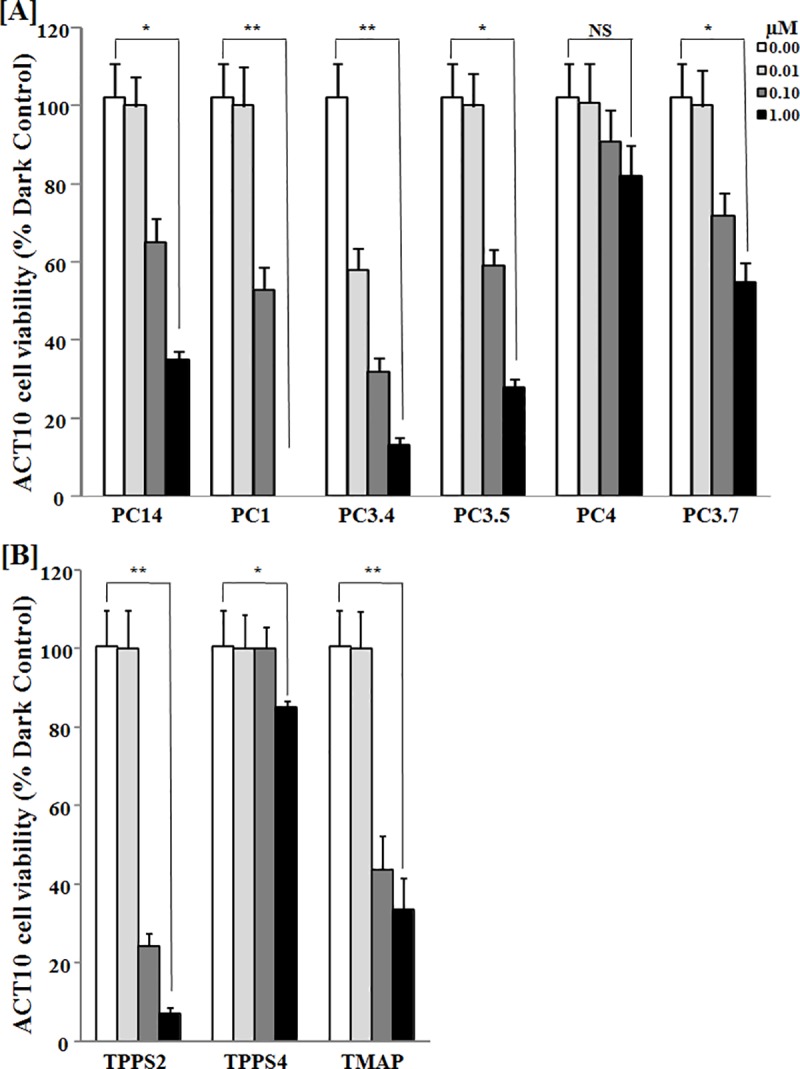
MTT reduction assays of *Aedes* cells for their viability *in vitro*, showing phthalocyanine **[A]** and porphyrin **[B]** concentration-dependent photo-inactivation. See Materials and Methods for experimental details. Briefly, Monolayers of ACT10 cells were exposed overnight in the dark to 0, 0.01, 0.1 and 1 uM photosensitizers (PS), as indicated in the legend of the graph. For each concentration of every PS used, one set was kept in the dark and the other set exposed to light. After further incubation overnight, all cell samples were processed in triplicate for the MTT reduction assay, as described. Cell viability is presented as % control by normalizing the values from light-exposed samples against those of the dark controls for each set of PS at all concentrations used. Data are presented for each PS in 4 bars with increasing shades of darkness, representing increasing PS concentrations from 0 to 1 uM, as indicated. **Note**: the PS concentration-dependent loss of cell viability by photo-inactivation in all cases. **NS**, Not significant; *, *p* = 0.01–0.05; ** *p* = 0.01–0.001.

Viability of *Aedes* cells was quantitatively assessed *in vitro* by MTT reduction assays after their loading in the dark with graded concentrations of PS in 10-fold serial dilutions (0, 0.01, 0.1, 1 uM) followed by light exposure (Fig **5**[A] and **5**[B]). PS concentration-dependent decrease in cell viability was significant for the larvicidal phthalocyanines, PC1, PC3.4, PC14 (Fig **5**[A]) and porphyrin derivatives, TPPS2, TMAP (Fig **5**[B]), but insignificant (Fig **5**[A] PC4) or marginally significant (Fig **5**[B] TPPS4) for non-larvicidal PS. Two non-larvicidal phthalocyanines (Figs **2 and 3**[A]) mediated photo-inactivation of *Aedes* cells *in vitro* (Fig **5**[A], PC3.5 and PC3.7).

### EC_50_ values of PS for light-activated larvicidal activities *in vivo* and photo-inactivation of *Aedes* cells *in vitro*

The EC_50_ values of representative PS in molar concentrations for light-activated 2^nd^ instar larvicidal activities showed the potency of newly discovered PS relative to the classic dyes of halogenated fluoresceins. The EC_50_ values of the effective PS (TPPS2, TMAP, PC1, PC3.4 and PC14) all fall in the nanomolar concentrations, ranging from 200–450 nM, being 4–10 times lower in value than that of rose bengal (1.9 uM) and 65–150 times more effective than cyanosine (30 uM). All five *in vivo* larvicidal PS are also effective *in vitro* for photo-inactivation of *Aedes* cells, their EC_50_ values determined *in vitro* being lower than those determined *in vivo* in most cases, i.e. TPPS2, TMAP, PC1, PC3.4 (Table 1, 2^nd^ Instar larva vs ACT10). *In vivo* ineffective PC remained mostly ineffective *in vitro*, e.g. PC4 and AlPhCl with few exceptions, e.g. PC3.5 (Table 1 ACT10 & C3/36 cells [22]).

**Table 1:** Estimated EC_50_ values of PS for light-activated larvicidal activities *in vivo* and photo-inactivation of *Aedes* cells *in vitro*.

Photosensitizer	EC_50_(μM)		
	2^nd^ Instar Larva	Cell line	
		ACT10^1^	C3/36^2^
Rose Bengal	1.9	ND	<50.0
Cyanosine	30.000	ND	<60.0
TPPS2	0.6	0.050	-
TMAP	0.2	0.040	-
PC1	0.4	0.100	-
PC 3.4	0.450	0.020	-
PC 3.5	>1.00	0.110	-
PC 4	>1.00	>1.00	>1.0
PC14	0.7	0.350	-
PhTHCl	>1.70	-	>1.7

^1^*Aedes aegypti* cell lines from Cindy Goodman, ARS-USDA, Colombia, MO

^2^*Aedes albopictus* clone C6/36 (ATCC CRL-1660) Data from Reference **[23]**.

ND, Not done

## Discussion

The major contribution of the present study is the identification of several novel PS (photosensitizers) as effective light-activated mosquito larvicides by screening a total of 16 different dyes from three chemical groups, i.e. phthalocyanines (PC), porphyrins and halogenated fluoresceins (Figs 1–3). The relative efficacy of these PS is validated by their side-by-side comparisons under identical laboratory conditions. Similar methodology used previously [18–21] was adopted here to simulate natural conditions, i.e. illumination of PS-preloaded larvae with white light of dim intensity for increasing time periods. The PDT (photodynamic therapy)-effective PS were identified by the increasing larvicidal activities with increasing PS pre-loading concentrations and increasing periods of illumination. The decrease in their effectiveness with increasing larva instars is an expected observation, consistent with the previous report [2].

Another contribution of this work is the use of insect cell lines to assess selected PS for PDT activities for cellular versus organismal comparison. All PS with larvicidal activities *in vivo* (Figs **2 and 3**) are also PDT-active against the mosquito cells *in vitro* (Figs **4 and 5**). Few PS with little or no larvicidal activities *in vivo* have intrinsic PDT activities against *in vitro* cultured cells (Fig **5**), e.g. PC3.5. The use of insect cell lines for screening PS is thus of value to eliminate those ineffective under both conditions. Comparative studies *in vitro* and *in vivo* are also expected to provide useful clues for developing PDI (photodynamic insecticides) based on PS structures versus activities. In addition, cultured insect cells are amenable to close examination under defined conditions, thereby facilitating the elucidation of cellular and molecular mechanisms of PS-mediated PDT. In the present study, we have initiated such investigation, showing the uptake of all effective PS by the mosquito cells (Fig **4**). This is consistent with the fact that such cellular event is known as a prerequisite for effective PDT activities [30, 32, 35]. Furthermore, the use of cell lines is expected to facilitate pre-screening of PS for PDT activities with discrimination against harmful pests, but not humans, pets, beneficial insects and environmentally friendly organisms, e.g. free-living protozoa, aquatic crustaceans and fish [2, 10, 37]. PC1/PC2, for example, are discriminatory PS, which mediate PDT to inactivate both mosquito cells and larvae (this study), but not mammalian cells [35].

Most significant is our finding of PC as a new group of PS with PDI activities, i.e. PC1/PC2, PC3.4, PC14 among a total of nine different PC examined, thereby adding new arsenals to advance PDI development. Of the five porphyrin-derivatives examined, TPPS2 and TMAP were also found as effective as the previously examined meso-substituted porphyrins of similar properties [20–21]. The EC_50_ values of the effective PS identified all fall in the nanomolar range for their larvicidal and cell-inactivation activities. These PS compare favorably in effectiveness to halogenated fluoresceins, especially cyanosine (Table 1). The effective PS identified provide lead compounds for structural modifications with potential to lower their EC_50_ values to the picomolar range, thereby rendering them field-deployable as PDI.

Evidence is provided, indicating that the effective use of PS for PDI is attainable via their chemical engineering. This is clearly suggested by our finding of only a handful of PS as PDT-active larvicides out of a total of 16 structurally different PS examined. In that regard, chemical synthesis of PC is of particular interest, as it is amenable to multiple structural modifications without losing its potency as PS for PDT activity. Engineering of PC by chemical synthesis has produced structurally versatile derivatives, i.e. the addition of side chains and ligands of variable structures and lengths to its peripheral rings at different positions and to the coordinating diamagnetic metals of different types, respectively [24–25, 38]. Such modifications of PC have been shown to increase its bio-availability. One example examined here is PC1/PC2, which are engineered to increase their cationicity by attaching two symmetrical mono- or di-amino groups to the coordinating Si, thereby enhancing their binding to the negatively charged cell surface to facilitate cellular uptake [25]. Another example is PC14, which is modified to prevent their stacking by attaching an uncharged bulky ligand to the coordinating Si, thereby enhancing the longevity of its solubility in aqueous environment conducive to cellular uptake [24]. As shown in the present study, both PC1/PC2 and PC14 were indeed taken up by mosquito cells into discrete intracellular structures, suggestive of endocytosis, consistent with the endosomal localization of these PC seen after their uptake by other eukaryotic cells [34]. It is not known if the endocytosis of these PC may result from their direct interaction with the cell surface or may be receptor-mediated via their initial binding to a protein ligand in the milieu [39, 40]. Most striking is the observation of dramatic differences seen in the anti-mosquito PDT-activities between PC3.4 and PC3.5 (Table 1), which differ only in the placement of the O-linked phenyl side chain in two peripheral rings from the alpha-position in PC3.4 to the beta-position in PC3.5 (Fig 1, PC3.4 vs PC3.5 Structure). How this slight shift in the position of a side chain renders PC3.5 totally ineffective *in vivo* (Figs 2 and 3) and less effective *in vitro* (Fig 5) is unclear. Clearly, the structural difference between PC3.4 and PC3.5 is too subtle to produce a significant difference in their cationicity and solubility for bioavailability. The finding thus underscores the potential of chemical engineering of PC as a new avenue worthy of further exploration for developing the next generation of effective PDI.

The use of PC as PDI is expected to broaden the range of PDT-targetable insects. This is inferred from the excitability of PC as a group for maximal PDT with red/infrared light, which is deep-penetrating through barriers, but invisible, in so far as is known, to most insects [40], except few beneficial groups, e.g. butter flies [41] and dragon flies [42]. In principle, PC is deliverable to insects via contact, ingestion, systemic routes and/or inhalation by using the methodology already available for other chemical insecticides. Delivery of PC by different routes is expected to affect the uptake of PC by different cell populations *en route* after the point of their entry, e.g. predominant sensitization of gut lining cells with PC after ingestion. Regardless of this variability, all PC-sensitized cells must be accessible to light as a mandatory step for target destruction notwithstanding the influence of its magnitude by a myriad of other factors. Sunlight is the most cost-effective, albeit chancy means of illumination for PDT [7, 18], having a polychromatic spectrum of wavelengths, of which the red range of ≥ 600 nm is known to penetrate deepest into human tissues through skin barrier [1, 10, 38]. By extrapolation, insects are likely more susceptible to PDT when sensitized with PC for red light excitation than those sensitized with other PS, e.g. most porphyrin derivatives and halogenated fluoresceins, which are excitable optimally by wavelengths of ~400 nm and 500–550 nm, respectively. Similarly, insects hidden in their natural habitats may be envisioned as more PDT-targetable when photo-sensitized with PC than with the other PS. While these assumptions must await experimental validation, different insects are predicted to vary greatly in response to PC-mediated PDT, considering their large differences in size, color and light translucency. In spite of this uncertainty, PC are favorably disposed to serve as effective PDI, taking into account the low EC_50_ values of their mosquito larvicidal activities, as shown in conjunction with their amenability to chemical engineering for enhancing their bioavailability, as discussed. One indisputable advantage of PC is their potential applicability day and night for PDT when used together with an artificial light source to emit red/infrared light. Since light of these wavelengths is invisible to most insects, it is not expected to cause evasive actions by them for avoidance, thereby exposing PC-sensitized ones to PDT for destruction. Thus, the use of PC and artificial lighting for PDT has the potential to substantially expand the rank of its targetable insects, irrespective of their nocturnal or diurnal phototropism, independent of sunlight for activation. While artificial light compares unfavorably to sunlight for the area of coverage, it is deployable by its strategic placement for effectiveness. This is the case at least for chemically attractable insects by lacing their food baits or odor lures with PC to sensitize the target insects under a red light-emitting source for their destruction by PDT.

Development of PC as PDI opens a new direction for insect control. It will complement the chemical approach to the synthesis of new insecticides and the genetic approaches to the production of pest-resistant crops [43, 44] or infertility-causing males for release to reduce or eliminate field populations [13–14]. The exceptional safety record and aversion to resistance represent the overriding advantages of PC-mediated PDT, compensating for its cumbersome requirement for light. Incorporation of PC-mediated PDT into integrated pest control programs is thus worthy of consideration to mitigate the persistent problem of the chemical approach and the uncertainty of the genetic approach [45].

## Supporting information

S1 Fig Differential sensitivity of 2^nd^ instar (Blank), 3^rd^ instar (gray) and 4^th^instar (Black) larvae of *Aedes aegypti* to light-induced inactivation mediated by [A] rose bengal (50 **μ**M) and [B] PC1 (1 **μ**M), [C] PC14 (1 **μ**M), [D] TMAP (10 **μ**M) and [E] TPPS2 (2 **μ**M). See Materials and Methods for experimental details. Briefly, groups of ~20 2^nd^instar (Blank), 3^rd^ instar (gray) to 4^th^instar (Black) larvae were exposed in the dark overnight to the photosensitizers (PS) at the concentrations as indicated. For both concentrations of each PS, one set of PS-exposed larvae was left in the dark and the other set exposed to white-light. Dead and live larvae were tallied hourly for 7 hours in all sets. Viability of the larvae was determined by visual inspection for their loss of motility and presented in % as the ratio of dead larvae in light-exposed versus dark conditions. Data presented represent results from three or more independent experiments.
(TIF)

## Abbreviations

PC: Phthalocyanines
PDI: Photodynamic insecticides
PDT: Photodynamic therapy/treatment
PS: Photosensitizers
