# Supplementary figures and images for: Novel phthalocyanines activated by dim light for mosquito larva- and cell-inactivation with inference for their potential as broad-spectrum photodynamic insecticides
Source: PLoS One. 2019 May 29;14(5):e0217355. doi: 10.1371/journal.pone.0217355 (PMC6541276; doi:10.1371/journal.pone.0217355)

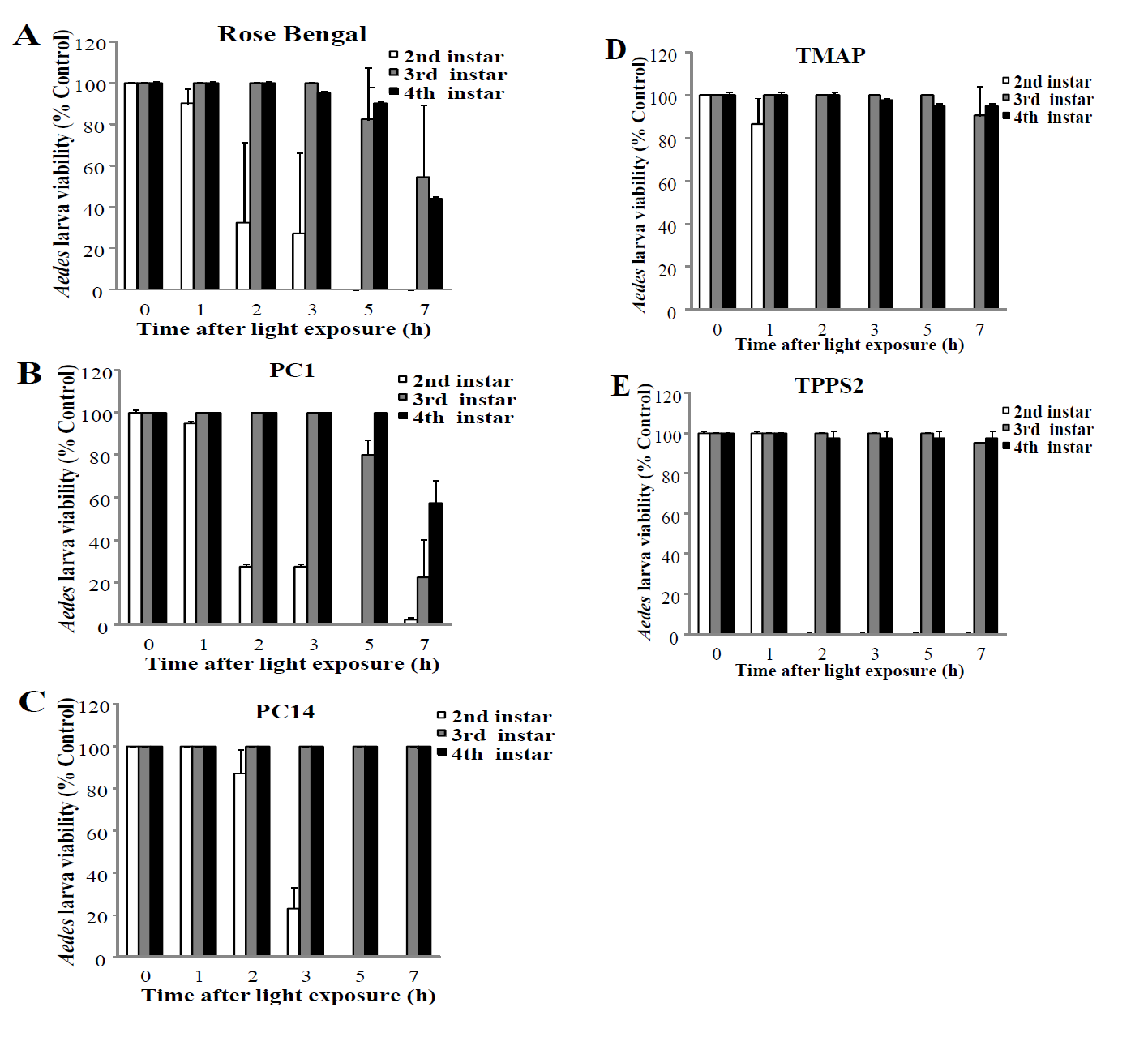

Supplement: S1 Fig — Differential sensitivity of 2nd instar (Blank), 3rd instar (gray) and 4thinstar (Black) larvae of Aedes aegypti to light-induced inactivation mediated by [A] rose bengal (50 μM) and [B] PC1 (1 μM), [C] PC14 (1 μM), [D] TMAP (10 μM) and [E] TPPS2 (2 μM). See Materials and Methods for experimental details. Briefly, groups of ~20 2ndinstar (Blank), 3rd instar (gray) to 4thinstar (Black) larvae were exposed in the dark overnight to the photosensitizers (PS) at the concentrations as indicated. For both concentrations of each PS, one set of PS-exposed larvae was left in the dark and the other set exposed to white-light. Dead and live larvae were tallied hourly for 7 hours in all sets. Viability of the larvae was determined by visual inspection for their loss of motility and presented in % as the ratio of dead larvae in light-exposed versus dark conditions. Data presented represent results from three or more independent experiments. (TIF) [file pone.0217355.s001.tif]
